# Microbial ecology in Latin America: underexplored opportunities of a transformative science

**DOI:** 10.1093/ismejo/wrag129

**Published:** 2026-10-06

**Authors:** L I Falcón, A Acosta-González, K Antúnez, L K Daille, C Dorador, J Duchicela, M A García-Amado, F Godoy-Vitorino, L C Mejía, L C Mendonca-Hagler, C Piccini, A A Pinto-Tomas, M M Rojas-Badía, M R Schiaffino, L E Servín-Garcidueñas, A F Solano-Arguedas, M Vásquez, L G Wall, M M Zambrano

**Affiliations:** Instituto de Ecología, Unidad Mérida, Universidad Nacional Autónoma de México, Ucú, Yucatán 97357, México; Grupo de Investigación en Bioprospección, Departamento de Procesos Químicos y Biotecnológicos, Facultad de Ingeniería, Universidad de la Sabana, Chía, 250047, Colombia; Laboratorio de Microbiología y Salud de las Abejas, Departamento de Microbiología y Centro de Investigaciones en Ciencias Ambientales, Instituto de Investigaciones Biológicas Clemente Estable. Montevideo, 11600, Uruguay; Centro GEMA-Genómica, Ecología & Medio Ambiente, Facultad de Ciencias, Universidad Mayor, Santiago, 8580745, Chile; Departamento de Biotecnología, Universidad de Antofagasta; Laboratorio de Complejidad Microbiana, Instituto Antofagasta, Universidad de Antofagasta, Antofagasta, 1270300, Chile; Departamento de Ciencias de la Vida y la Agricultura, Universidad de las Fuerzas Armadas-ESPE, Sangolquí, 171103, Ecuador; Laboratorio de Fisiología Gastrointestinal. Centro de Biofísica y Bioquímica. Instituto Venezolano de Investigaciones Científicas (IVIC), Caracas, 1204, Venezuela; Microbiology and Immunology Department, University of Puerto Rico School of Medicine, Medical Sciences Campus, San Juan, PR 00926, United States; Grupo de Interacciones en Ecosistemas, División de Biodiversidad, Instituto de Investigaciones Científicas y Servicios de Alta Tecnología (INDICASAT AIP), Ciudad del Saber, Panamá, 07144, República de Panamá; Instituto de Microbiologia Professor Paulo de Góes, Universidade Federal do Rio de Janeiro, Rio de Janeiro, Rio de Janeiro, 21941-853, Brasil; Departamento de Microbiología, CICA-Instituto de Investigaciones Biológicas Clemente Estable. Avenida Italia 3318. Montevideo, 11600, Uruguay; Departamento de Bioquímica, Escuela de Medicina y Centro de Investigación en Biología Celular y Molecular, Universidad de Costa Rica, San José, 11501-2060, Costa Rica; Grupo de Ecología Microbiana, Dpto. de Microbiología y Virología, Facultad de Biología, Universidad de La Habana, La Habana, 10400, Cuba; Departamento de Ciencias Básicas y Experimentales, Universidad Nacional del Noroeste de la Provincia de Buenos Aires (UNNOBA). Centro de Investigaciones y Transferencia del Noroeste de la Provincia de Buenos Aires (CITNOBA)—UNNOBA-UNSAdA-CONICET, Junín, Buenos Aires, B6000, Argentina; Laboratorio de Microbiómica, Escuela Nacional de Estudios Superiores Unidad Morelia, Universidad Nacional Autónoma de México, Morelia, Michoacán, 58190, México; Unidad de Recursos Forestales (Reforesta), Instituto de Investigaciones en Ingeniería (INII) and Escuela de Química, Universidad de Costa Rica, San José, 11501-2060, Costa Rica; Facultad de Ciencias Biológicas, Pontificia Universidad Católica de Chile, Santiago, 8331150, Chile; Laboratorio de Bioquímica y Biología de Suelos, Centro de Bioquímica y Microbiología del Suelo, Universidad Nacional de Quilmes, Bernal, B1876BXD, Argentina; Corpogen Research Center, Bogotá, Colombia; Dirección de Investigaciones y Transferencia de Conocimiento, Universidad Central, Bogotá, 110311, Colombia

**Keywords:** ISME Lat, capacity building, biodiversity hotspots, environmental microbiology, scientific equity, Global South, microbial communities

## Introduction to the region

Latin America—part of the Global South—hosts some of the planet’s most biodiverse ecosystems. These biodiversity “hotspots” are home to unique and vital habitats for our planet’s health and function, including the Amazon, Moskitia, Indio Maíz-Tortuguero, La Amistad, Darién, Chocó, Chaco, Orinoco and Selva Maya rainforests, the Andes, the Atacama Desert and the second-largest coral barrier [1]. Latin America harbors roughly 40%–60% of the world’s terrestrial biodiversity and a substantial portion of its freshwater, mangrove, and forest ecosystems [1, 2] including ancient microbial communities—such as microbialites, and highly diverse soil and oceanic microbiomes that harbor a vast, often unknown, microbial life [3]. These microbial systems [4] play key roles in resilience and ecological restoration where the unprecedented diversity creates fascinating natural laboratories to study ecosystem functions, nutrient cycles, carbon sequestration, climate regulation, and adaptations to extreme environments and anthropogenic pressures, as well as a superb potential for bioprospection [5]. The region’s biological and cultural diversity, coupled with its scientific talent, positions Latin America as a key player in addressing environmental challenges including climate change, biodiversity loss, and ecosystem degradation. Yet, Latin America currently leads the International Union for Conservation of Nature (IUCN) Red List, with more than 4000 species classified as threatened [1].

Latin America is home to over 800 indigenous cultures, which have different approximations and connections to nature ([1]; https://www.alianzamesoamericana.org), evident in the wealth of plant domestications (corn, potatoes, beans, cacao, squash, and cotton to name a few) [1]. The oldest academic institutions are also in the Americas, first established in 1518 in República Dominicana and studies in natural sciences have a long-standing tradition in the region, among which microbial ecology has gained momentum and become more formally organized since the late 20th and early 21st centuries. Several institutions offer programs ranging from bachelor’s to master’s and doctoral degrees in microbiology, covering fields in human and animal health, ecology, and biotechnology.

Despite its extraordinary natural and cultural richness, Latin America has been shaped by a shared history of colonization, economic dependence, extractivism, and persistent challenges in areas such as health care and access to education. European colonialism and the introduction of different animal husbandry and exploitative agricultural practices changed human-nature relations that have transgressed the planetary biodiversity boundaries [1]. Constant structural barriers—insufficient and unstable funding, uneven research infrastructure, and language-based inequities—continue to limit scientific research productivity and reduce the global visibility of scholarship from the region [6–9]. Latin America has been underrepresented in global microbial ecology research. Only in recent years has the region become more visible [10]. However, the limited presence of Latin American microbial ecology research in international conferences and scientific publications underscores the need to enhance the region’s visibility and strengthen global collaborations [11]. These obstacles are further compounded by the prevalence of “helicopter research,” in which foreign researchers lead studies in the region with minimal local collaboration or capacity building, even without adequate national permissions to access genetic or biochemical resources in complete flagrance of Latin American countries’ biodiversity policies [1, 8, 12–14]. Funding gaps severely restrict the Global South’s scientific capabilities and impact, affecting everything from conducting comprehensive research to attending scientific meetings. Empirical evidence shows that high conference costs and insufficient concession policies systematically exclude scientists from low- and middle-income regions, reinforcing disparities in visibility, collaboration networks, and authorship representation [15], underscoring the need for targeted policies that promote equitable participation and inclusive knowledge production in microbial ecology.

At the same time, the accelerating pressures from climate change, species extinction, and persistent social and environmental inequities demand that microbial science in Latin America moves beyond descriptive inventories towards transformative research that not only generates data but also produces solutions tailored to local needs, catalyzing change and involving stakeholders. For decades, microbial ecologists across the region have made important scientific contributions that have triggered social initiatives for biodiversity conservation and recognition, despite chronic limitations in funding and infrastructure. Documenting and understanding the immense microbial diversity embedded within Latin America’s biodiverse ecosystems has been a monumental undertaking for the many researchers who have devoted their careers to this effort. Continued research and collaboration are essential to unravel the intricate microbial interactions and functional pathways that underpin ecosystem services and to confront urgent challenges such as climate change and biodiversity loss. The initial step toward addressing these challenges is to formally acknowledge and thoroughly diagnose this inequality, while fully embracing the geographical and cultural diversity present [16]. Addressing these gaps through strengthened regional collaborations, innovative research networks, and inclusive capacity-building initiatives offers transformative opportunities to advance microbial science, i.e. both globally impactful and locally relevant. This perspective presents the ISME Lat biennial meetings as a catalytic opportunity to help establish the region as a hub for microbial ecology research, with the potential to inspire similar initiatives in other underrepresented regions.

## ISME Lat biennial meetings

Catalyzed by the interest of ISME to involve more microbial ecologists from Latin America, the regional ambassadors jointly organized a biennial meeting (ISME Lat) beginning in 2019. These meetings have been successful in large part due to the shared history, interests, and culture among participants. Since its inception, ISME Lat has strengthened regional collaboration and raised the visibility of local researchers, becoming a key initiative for advancing microbial ecology across Latin America. The latest installment took place in August 2025 in Mérida, Yucatán, México (Fig. 1), following previous events held in Chile (2019), Colombia (online, 2021), and Argentina (2023). The main goal of ISME Lat meetings is to disclose the work being done and connect the large Latin American network working on all aspects of microbial ecology. The fourth ISME Lat event brought together over 500 participants from 24 countries, encompassing a broad geographic and professional diversity that created unprecedented opportunities for networking across countries (Fig. 2). Registration categories highlighted early-career scientists and student representation. Young scientists’ participation was supplemented by professionals, early-career researchers, regional delegates, and volunteers. It successfully drew the attention of young participants, including first-time ISME participants. The ISME Ambassador Program has expanded the Latin American leadership community in microbial science through trans-border collaborations, increased visibility of regional science, and contributed to mentoring of early-career researchers. The group is multidisciplinary and represents consolidated research programs with a strong social commitment. Ambassadors (https://isme-microbes.org/community/isme-ambassadors/) are involved in organizing ISME Lat meetings and workshops and have been fundamental in creating this community.

**Figure 1:**
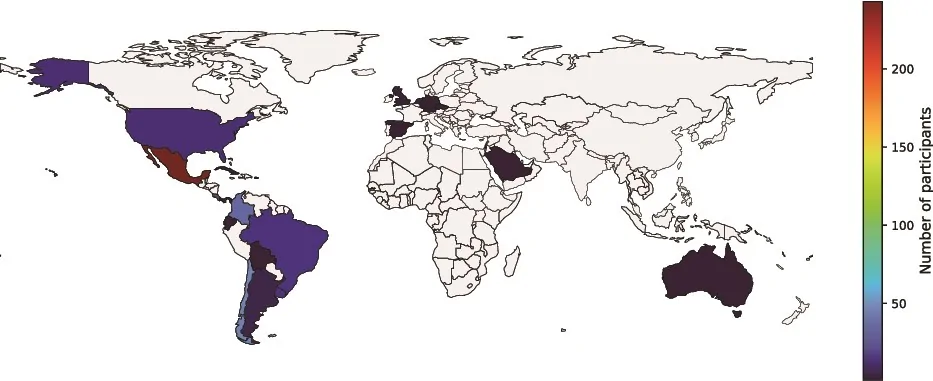
Choropleth map of participant counts by country. Cool-to-warm colors reflecting increasing values. Countries with no participants appear in light gray for reference.

**Figure 2:**
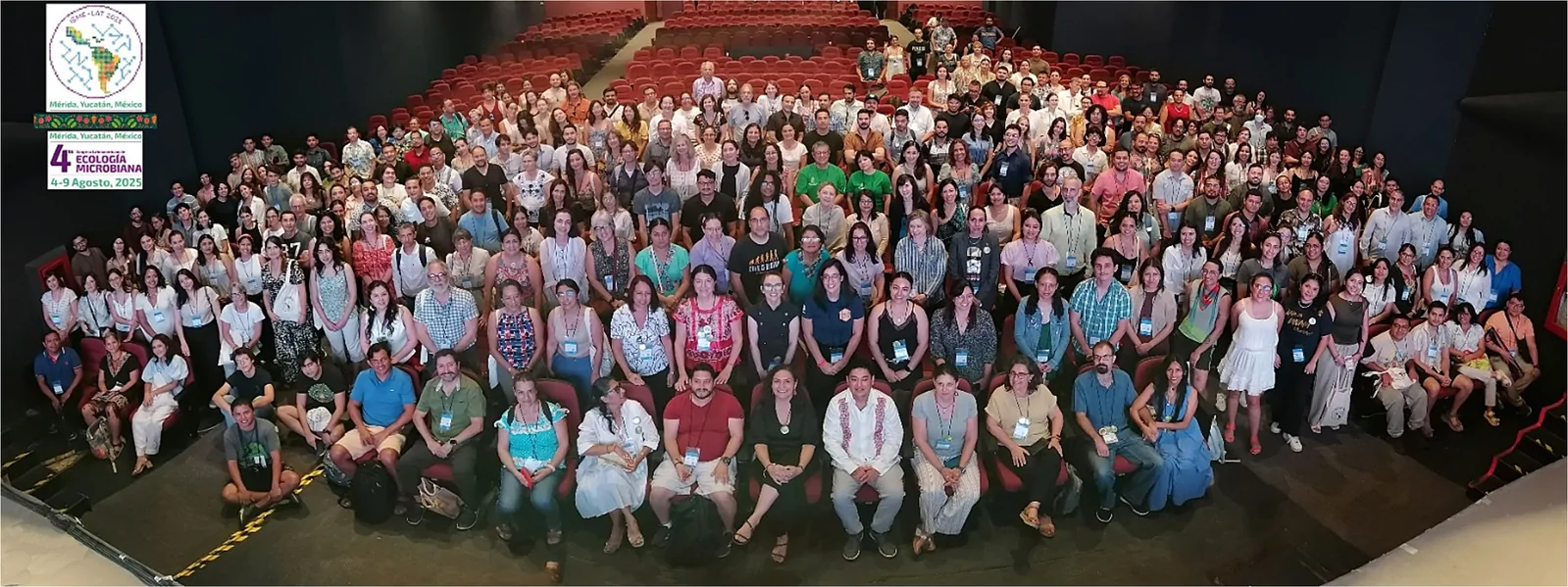
Group photo of the fourth ISME Lat, taken on 7 August, 2025.

The meeting’s guiding theme, “With the Community, For the Community,” (“de la comunidad, para la comunidad”) functioned not merely as a slogan but as a foundational ethos that informed every aspect of the event—from community-centered science festivals to participatory poster sessions. The program featured four keynote addresses by distinguished leaders in the field: Antonio Lazcano (México) reflected on the future of scientific enterprise in the Americas; Esperanza Martínez Romero (México) examined the intricate symbioses between crops and their microbiomes (symbiovar); Antonio González Peña (USA–México) explored data science platforms like QIITA advancing microbiome research and the importance of metadata; and Osvaldo Ulloa Quijada (Chile) shed light on the microbial life of the deep sea. Together, these plenaries set an exceptional scientific tone while firmly situating the conference within broader socio-political and environmental contexts.

The meeting was organized along three axes: Environmental Microbial Ecology; Microbiome Ecology; and Microbes and Biotechnology, and served both as an academic gathering and as an attempt toward collective action to enhance Latin American microbial ecology through open engagement, science outreach, and capacity building. Together, these thematic axes shaped the scientific discourse and facilitated a comprehensive examination of microbial life across diverse systems and scales.

The first axis, Environmental Microbial Ecology, encompassed sessions on soils and rhizobiomes, epicontinental aquatic systems, oceanic environments, extremophiles, and the microbial ecology of Antarctica. It also included specialized topics such as virus ecology, models of early life and astrobiological systems, and the use of bioinformatic tools to understand complex microbial systems. México has a strong tradition of studying the early evolution of life. Throughout Latin America, there are critical sites where astrobiological model ecosystems are explored, including extremophilic environments, microbial mat, and microbialite ecosystems. Antarctic research is also a key feature in the region, as several Latin American countries have scientific bases and research programs. Since the 1970s, there has been ample research on soil microbial ecology, with agroecological practices that conserve biodiversity, such as the milpa, the ancient Mesoamerican polyculture farming technique based on growing over 30 species, including corn, squash, and beans in parallel, receiving attention. Microbial ecology studies of mangroves are another trending topic of great socio-environmental relevance of a highly threatened yet productive ecosystem in tropical coastal areas.

The second thematic axis, Microbiome Ecology, examined the intricate connections between microbes, their hosts, and health. Sessions addressed a wide range of topics, including medical microbiome initiatives, host–microbiome interactions, symbiotic relationships, the ecology of emerging pathogens with implications for future pandemics, and the contributions of chemical ecology to microbial communication and function. Discussions also delved into insect–plant symbioses, rhizobiome dynamics, the cacao microbiome, and the importance of fermented ancestral foods, highlighting their relevance to reverse malnutrition, disease processes, and microbial dysbiosis.

The third axis, Microbes and Biotechnology, highlighted the technological frontiers of microbial research. Sessions covered novel techniques for microbial study, the biotechnology of natural products and drug discovery, the use of heterologous hosts as cell factories, as well as environmental and food biotechnologies, including agricultural applications, bioremediation, and microbial fermentation.

ISME Lat meetings play a pivotal role in promoting inclusivity and strengthening regional scientific initiatives. A hallmark of ISME Lat is its sustained commitment to supporting emerging researchers. Through lightning talks, an early-career panel, and other tailored activities, students and postdoctoral fellows occupied a central place in the program. The ISME ECS program, in particular, provided a supportive and constructive space for candid discussions about the challenges of publishing, securing funding, cultivating effective mentorship, and attending to mental health within academic environments [6, 7]. The ISME ECS Committee actively promotes initiatives specifically designed to strengthen interaction, networking, and professional development opportunities and provides a space for exchanging and following up on the challenges and opportunities in the region [17].

Besides the academic program, the ISME Lat meetings host a variety of outreach activities, including community workshops, open-audience talks on a variety of subjects (“What will the next pandemic be?”; “Studying our microbial planet”; “On the origins of life”); and a series of videos and a photographic exhibition showcasing microbial ecology research in different environments. ISME Lat meetings have three official languages: Spanish, Portuguese, and English, which has triggered the participation of many students from different countries. It is suggested that written material (posters/slides) are in English, but participants can speak in any of these languages.

## Research frontiers in Latin American microbial ecology

Microbes are key to the conservation of ecosystems, sustainable development, and adaptation to climate change. Their relevance is reflected in the many regional initiatives and projects that involve microbiology. The new IUCN Microbial Conservation Specialist Group includes Latin American experts who have spearheaded initiatives on the role of microbes in ecosystem health and function. Major efforts have focused on the complexity of lithium mining and protecting high-altitude saline lakes as “green technologies” have driven an eightfold increase in lithium production and groundwater overextraction generating “microdisasters” [3, 18]. Similarly, in México, threats to microbialite reefs, rock-like structures formed by microbial communities, driven by declining water quality, massive tourism, and groundwater overextraction have triggered conservation strategies, including efforts towards their legal recognition. Several collaborative initiatives involving systematic and rigorous sampling and analysis have also emerged, aimed at strengthening capacity building and connecting researchers and students across countries. Some of these include:

The Latin American Collaborative Network for Aquatic Microbial Ecology (μSudAqua), a regional initiative that promotes coordinated research on microbial communities across diverse aquatic systems (https://microsudaqua.netlify.app/).

The South American Mycorrhizal Research Network (SAMN) (https://southmycorrhizas.org/), fosters collaboration, and research on mycorrhizal fungi.

LatinBiota (https://github.com/alexmsalmeida/latinbiota) is a central repository of gut microbiome datasets, with scripts and workflows for parallel analysis.

CABANA (https://cabana.network/) is a consortium of Latin American institutions that supports capacity-building and data-driven biology research. It has addressed the training needs in the biological and biomedical sciences along three axes: communicable diseases, sustainable food production, and biodiversity protection.

There are also several culture collections and genomic libraries stored in academic institutions in the region. All of these initiatives demonstrate a growing recognition of the relevance of microbes and the need for coordinated efforts to promote scientific excellence and results that benefit society through sustainable development strategies.

## Looking forward: a vision for Latin American microbial ecology

Initiatives such as the ISME Lat biennial meetings, Latinbiota, CABANA, μSudAqua, and SAMN foster strategies to build on shared scientific infrastructure, including regional reference bioreservoirs, harmonized metadata, and quality controls. Yet, none of these have secured public or private mid to long-term funding. ISME Lat is triggering collaborations that aim to conduct integrative studies to plan conservation strategies of unique ecosystems based on their microbial diversity, including conservation of fecal microbiomes to protect beneficial microbes representing different lifestyles and cultures. Also, the meetings aid in getting together and consolidating the teams working on the fundamental challenges such as: integrating time-series along regional gradients, protecting and managing natural resources where sustaining water, soil, air quality is fundamental to securing food and health of human populations and ecosystems. The recent establishment of Centers of Biomedical Research Excellence (COBRE), launched in Puerto Rico as the Center for Microbiome Sciences (PR-CMS) [11], marks a pivotal moment for capacity-building in Latin American microbial research. As the first center of its kind in the Caribbean and funded by the NIH, the PR-CMS provides essential sequencing and bioinformatics cores, mentorship opportunities for early-career investigators, and infrastructure that supports interdisciplinary, regionally relevant research, while promoting greater participation and access to otherwise underrepresented partners. More initiatives like COBRE are needed.

Without mid- to long-term planning supported by robust public–private partnerships and memoranda of understanding across countries, research and development initiatives in microbial ecology remain confined largely to the capacities of local academic institutions. This creates a structural bottleneck in which universities shoulder responsibilities that should be shared across sectors, including technological innovation and the translation of scientific discoveries into societal impact. In the absence of governmental commitment and private-sector investment, institutions struggle to secure the sustained funding, infrastructure, and interdisciplinary collaboration required to advance high-impact research. As a result, opportunities for scaling innovations, supporting early-career scientists, and integrating science into policy and industry remain limited, constraining the region’s ability to compete globally and to leverage microbial science for environmental, productive, and public health solutions.

## Conclusions

ISME Lat meetings are catalyzing a new era for microbial ecology in Latin America and represent a model for inclusive scientific governance, a testbed for equitable international collaboration, and a driver of globally relevant research. Yet, Latin America is only one of the regions impacted by Global South conditions. Diversity, equity, and inclusion practices have been fundamental for the access and academic development of under-represented students and researchers through programs, collaborations, and training in developed countries. Now, many of these initiatives built over decades are being threatened, affecting science worldwide [19, 20]. The ISME Lat community-driven model could therefore be adapted and replicated in other underrepresented regions (e.g. Africa or South/Southeast Asia), positioning ISME as a potential blueprint for inclusive scientific development worldwide: connect with researchers in your region, plan meetings, find common topics, and get together in different countries, being sensitive to regional necessities (e.g. language barriers, funding, training needs). Microbes have taught us that there is no single species that can live alone—cooperation, facilitation, and sensitivity towards our neighbors goes a long way in nature—let's practice this in our academic trajectories and development.

ISME regional meetings contribute to the global ISME community, beyond increased representation and participation of a dynamic, talented scientific community from regions with unmatched biodiversity that are increasingly recognized for its leadership, innovation, and commitment to equity. Yet, persistent inequities—financial, gender, infrastructural, linguistic, and geopolitical—continue to restrict scientific potential. Strengthening regional networks, expanding training opportunities, and securing long-term collaboration frameworks will be essential for transformation. Latin American microbial ecologists are no longer peripheral observers; the conversations and collaborations sparked at ISME Lat meetings—and sustained through the ambassador program—underscore that the region’s ecological diversity is mirrored by its scientific talent and the innovative approaches driving high-quality research. ISME’s continued engagement is vital for ensuring that Latin American perspectives and contributions remain integral to the global scientific agenda. By providing a platform where microbial science from the region can stand on equal footing with work from around the world, ISME Lat meetings contribute meaningfully to shape the future of research in Latin America. By including students, early-career investigators, senior scientists, and community representatives, these meetings highlight cutting-edge advances in microbial ecology while embedding them within a broader commitment to equity and social responsibility. ISME Lat meetings also offer critical opportunities for engagement through workshops, webinars, and regional networking, thereby strengthening scientific capacity and collaboration across the continent.

Latin American scientists are highly trained, deeply motivated, and increasingly influential contributors to global microbial science. They are reshaping the field by integrating biodiversity-driven inquiry, technological innovation, and a strong ethic of social and environmental responsibility. Their voices are gaining prominence and advancing the frontiers of microbial ecology. As in many parts of the world, researchers in Latin America face entrenched structural challenges, including chronic underfunding, uneven access to research infrastructure, and limited capacity to recruit and retain new scientific talent at a consistent pace. They also continue to navigate the legacy of extractive and inequitable research practices. To transform Latin American microbial ecology requires more than the expansion of technical capacities and to address the historical structures shaping knowledge production in the region. It is essential to move from a model characterized by externally defined scientific agendas, infrastructure, and funding, toward an approach based on regional scientific sovereignty. This implies not only strengthening local infrastructure and sustainable funding mechanisms, but also redefining research priorities based on territorial issues, promoting equitable governance schemes for data and biological resources, and consolidating international collaborations under principles of reciprocity and co-leadership. Furthermore, if the Latin American scientific community continues to measure scientific success solely by publishing in journals in the Global North, the system will not change. Therefore, the metrics used to measure scientific productivity should be broadened to consider the impact on public policy, local applications, regional networking ability, and technology transfer at the local or regional level. Likewise, integrating local knowledge and cultural practices into the study of microbiomes represents a key dimension for advancing toward a more inclusive and contextualized science. Together, these changes would allow us to overcome persistent extractive practices and position Latin America not only as a data provider, but also as a generator of strategic knowledge with scientific, environmental, and social impact on a global scale. The experience of ISME Lat meetings demonstrates that these barriers can be addressed through regional solidarity, cross-border collaboration, and strategic engagement with international partners.

The region’s scientific community has demonstrated through the organization of ISME Lat meetings its ability to unite on its own terms, articulate its own priorities, and develop research agendas grounded in both global scientific standards and local realities. The microbial ecology of Latin America is not a peripheral chapter in the international narrative. It is a driving force that shapes research directions, methodological innovations, and policy-relevant science. Those committed to the future of microbial ecology are encouraged to join and strengthen this collective effort. The momentum generated by the ISME Lat meetings demonstrates that a more equitable, collaborative, and impactful future of microbial ecology is both possible and marks the beginning of a new era in which Latin American microbial science will shape global scientific discourse for decades to come. See you in Costa Rica, July 2027, for the fifth ISME Lat!

## Data Availability

Data sharing not applicable to this article as no datasets were generated or analyzed during the current study.
